# Supplementary material for: Lead-Free Perovskite Homojunction-Based HTM-Free Perovskite Solar Cells: Theoretical and Experimental Viewpoints
Source: Nanomaterials (Basel). 2023 Mar 8;13(6):983. doi: 10.3390/nano13060983 (PMC10054775; doi:10.3390/nano13060983)
Supplement: Supplementary file 1 [file nanomaterials-13-00983-s001.zip › nanomaterials-2286845-supplementary.pdf]

Lead-free perovskite homojunction-based HTM-free perovskite solar cells: Theoretical and experimental viewpoints

Sajid Sajid[1,2], Salem Alzahmi[1,2,*], Imen Ben Salem[3], Jongee Park[4], and Ihab M. Obaidat[2,5,*]

1 Department of Chemical & Petroleum Engineering, United Arab Emirates University, Al Ain P.O. Box 15551, United Arab Emirates
2 National Water and Energy Center, United Arab Emirates University, Al Ain P.O. Box 15551, United Arab Emirates
3 College of Natural and Health Sciences, Zayed University, Abu Dhabi P.O. Box 144534, United Arab Emirates
4 Department of Metallurgical and Materials Engineering, Atilim University, Ankara 06836, Turkey
5 Department of Physics, United Arab Emirates University, Al Ain P.O. Box 15551, United Arab Emirates
* Correspondence: iobaidat@uaeu.ac.ae; s.alzahmi@uaeu.ac.ae

Supporting Information

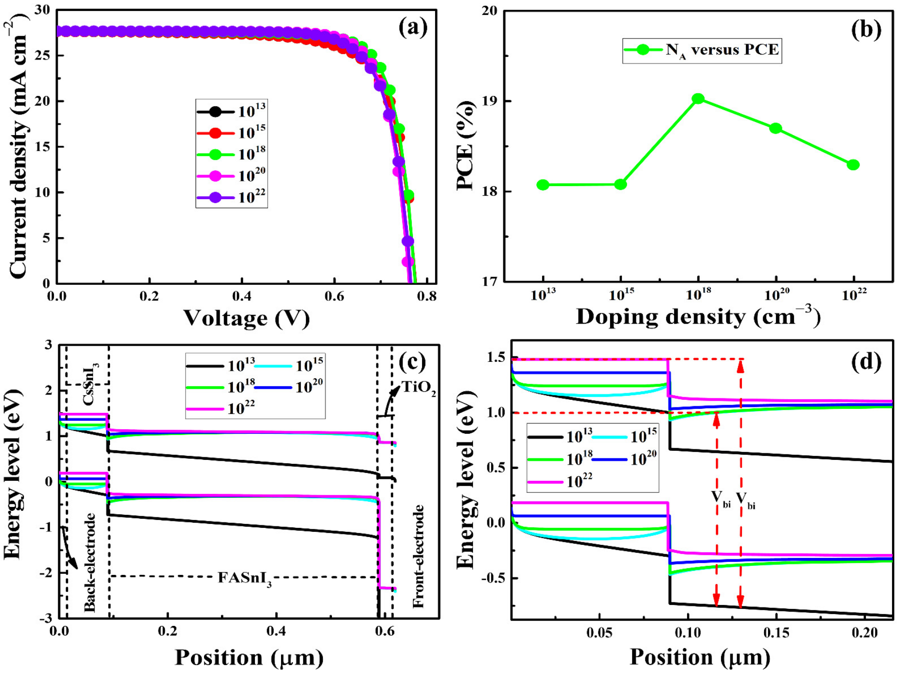

Figure S1. The current-voltage (J-V) characteristic curves (a), power conversion efficiencies (PCEs) (b), energy levels (c) and zoomed-in energy levels (d) of the as-simulated homojunction-based HTM-free PSCs as a function of doping density of $CsSnI_3$. Note that the $FASnI_3/CsSnI_3$ region is where the zoomed-in energy levels were taken.

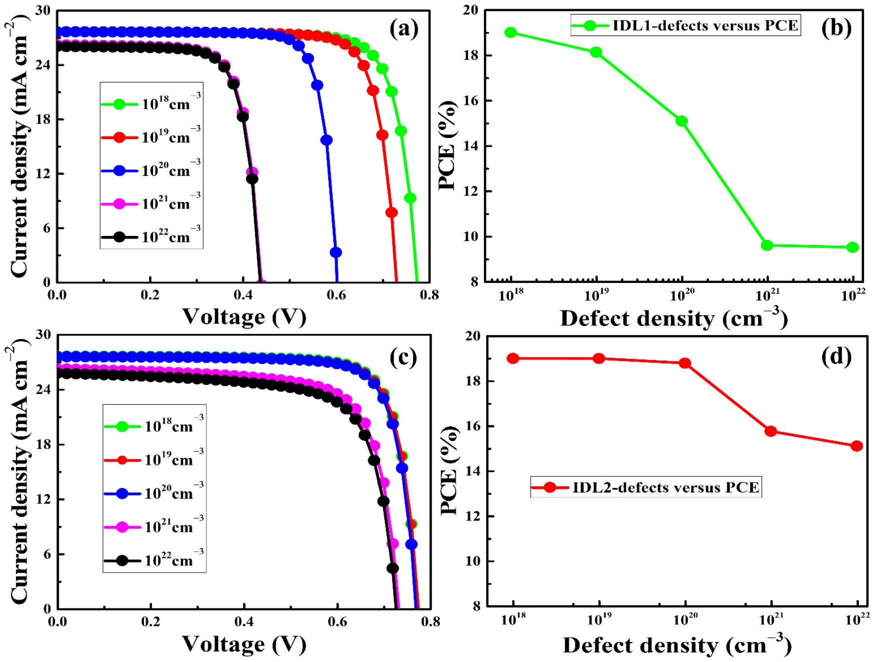

Figure S2. The current-voltage (J-V) characteristic curves and power conversion efficiencies (PCEs) of the as-simulated homojunction-based HTM-free PSCs as a function of defect density in IDL1 (a, b) and IDL2 (c, d).

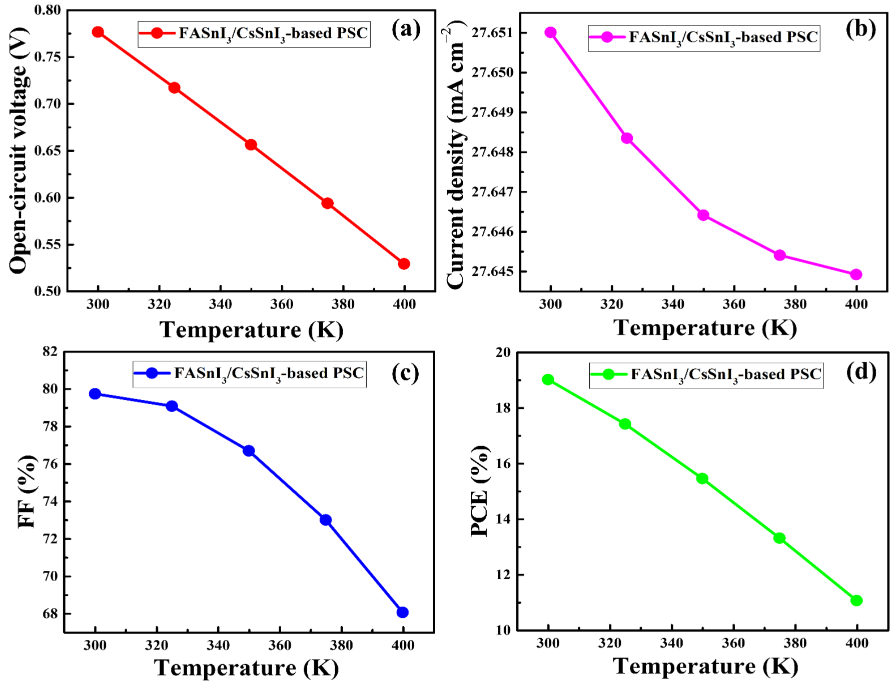

Figure S3. Photovoltaic performance of the as-simulated homojunction-based PSCs as a function of temperature.

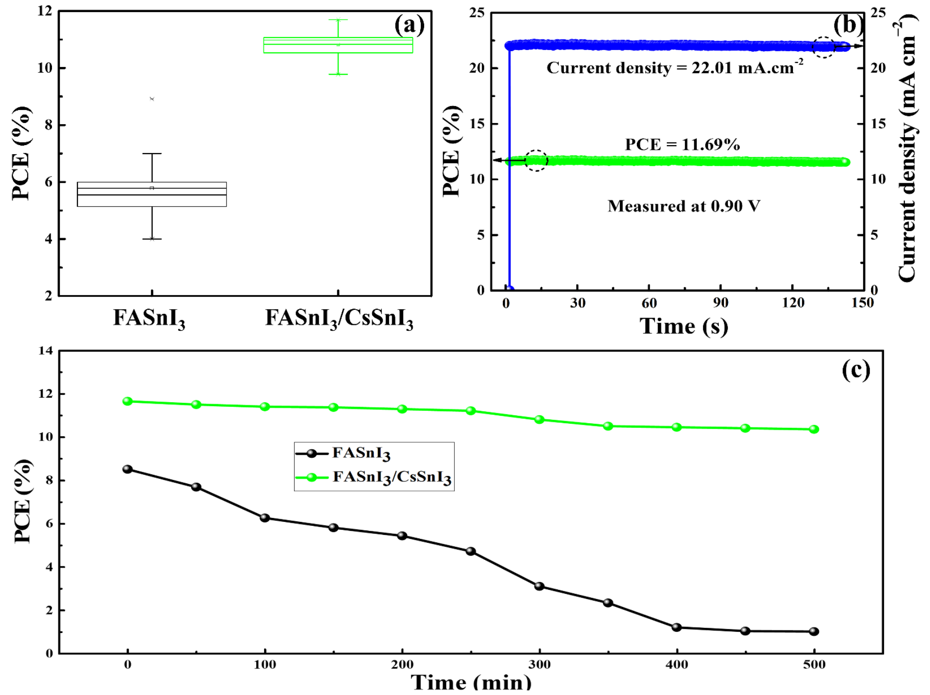

Figure S4. Statistics of the HTM-free PSCs with $FASnI_3$ or $FASnI_3/CsSnI_3$ (a). Efficiency and current density of $FASnI_3/CsSnI_3$ homojunction-based HTM-free PSC at maximum power point tracking (b), long-term stability of the $FASnI_3$-based devices and $FASnI_3/CsSnI_3$-based devices at 25℃ in air (c).
